# Diagnostic Performance of Selected Baseline Electrocardiographic Parameters for Prediction of Left Ventricular Remodeling in Patients with ST-Segment Elevation Myocardial Infarction

**DOI:** 10.3390/jcm10112405

**Published:** 2021-05-29

**Authors:** Michał Kasprzak, Tomasz Fabiszak, Marek Koziński, Jacek Kubica

**Affiliations:** 1 Department of Cardiology and Internal Medicine, Collegium Medicum, Nicolaus Copernicus University, ul. M. Skłodowskiej-Curie 9, 85-094 Bydgoszcz, Poland; tfabiszak@wp.pl (T.F.); jwkubica@gmail.com (J.K.); 2 Department of Cardiology and Internal Medicine, Medical University of Gdansk, ul. Powstania Styczniowego 9B, 81-519 Gdynia, Poland; marekkozinski@wp.pl

**Keywords:** myocardial infarction, ECG, risk stratification, left ventricular remodeling

## Abstract

**Objective:** To evaluate the diagnostic performance of selected baseline electrocardiographic (ECG) parameters as predictors of left ventricular remodeling (LVR) in patients with a first ST-segment elevation myocardial infarction (STEMI) treated with primary percutaneous coronary intervention (PCI). **Methods:** The study was performed as a single-center cohort study, with 249 patients (74.7% males) included in the final analysis. Nine baseline ECG parameters were evaluated, with respect to occurrence of LVR 6 months after STEMI (defined as an echocardiography-assessed relative >20% increase in end-diastolic left ventricular volume compared with the value at discharge from hospital). **Results:** The baseline ECG predictors of LVR, identified in univariate analysis, included the number of leads with ST-segment elevation (odds ratio (OR) 1.19, 95% confidence interval (CI) 1.03–1.38, *p* = 0.0212), number of leads with Q-waves (OR 1.21, 95% CI 1.07–1.37, *p* = 0.0033), sum of ST-segment elevation (OR 1.04, 95% CI 1.00–1.08; *p* = 0.0253) and maximal ST-segment elevation (OR 1.14; 95% CI 1.00–1.29; *p* = 0.0446). When added to demographic, clinical and angiographic data, the number of leads with ST-segment elevation (OR 1.17, 95% CI 1.01–1.36; *p* = 0.0413), number of leads with Q-waves (OR 1.15, 95% CI 1.01–1.32; *p* = 0.0354) and the sum of ST-segment elevation (OR 1.04, 95% CI 1.00–1.08; *p* = 0.0331) successfully predicted development of LVR in multivariate logistic regression models. However, after inclusion of biochemical data in multivariate models, none of the electrocardiographic parameters, but increasing body weight, TIMI flow after PCI < 3 and higher maximal values of myocardial necrosis biomarker, was independently associated with the occurrence of LVR 6 months after STEMI. **Conclusions:** Our study demonstrates modest utility of pre-reperfusion ECG for the prediction of LVR occurrence after six months of STEMI.

## 1. Introduction

Despite more than a hundred years since introduction to clinical practice, electrocardiography (ECG) remains one of the basic modalities in cardiac diagnostics [1]. ECG plays a key role in risk stratification and selection of treatment strategies in patients with acute coronary syndromes (ACS) [2]. Not only does it form the basis for the division of the latter into ACS with and without persistent ST-segment elevation, implicating the manner and timing of the management, it also allows stratification of short- and long-term risk [3,4,5].

One of the long-term consequences of myocardial infarction (MI) is the occurrence of left ventricular systolic dysfunction (LVSD). It depends mainly on the extent of the post-MI necrosis, presence of frozen myocardium and occurrence of left ventricular remodeling (LVR) [6,7]. The latter begins in the first hours of MI and can last for weeks or even months. LVR consists in left ventricular dilation and hypertrophy with focal myocardial fibrosis, leading to transformation from the natural ellipsoidal shape of the left ventricle to the spherical one, and in some cases also the formation of a post-MI aneurysm of the left ventricle [6,7,8,9,10]. It should be noted that LVR may occur despite successful revascularization and maintenance of normal global and segmental left ventricular systolic function and may affect up to 30% of patients after MI [11]. The most frequently used method of assessment of LVSD and LVR presence is echocardiography. Commonly used echocardiographic parameters describing left ventricular systolic function and its remodeling are left ventricular ejection fraction (LVEF) and left ventricular end-diastolic volume (LVEDV) [10]. The presence of LVR carries an increased risk of death from cardiovascular causes and development of heart failure [7,10,11,12,13]. In the 5-year observation, it is associated with a nearly three-fold increase in mortality and a nearly two-fold higher incidence of serious cardiovascular events including death, non-fatal MI and heart failure requiring hospitalization [11].

There are many reports assessing the prognostic value of electrocardiographic parameters on the occurrence of LVSD [3,4,14,15], while literature data on the occurrence of LVR are scarce. In addition, the most important and largest of the studies confirming the importance of ECG for risk stratification in ST-segment elevation myocardial infarction (STEMI) were carried out in the era of thrombolytic therapy [14,15]. Since then, STEMI treatment has undergone revolutionary changes with the introduction of effective and safe reperfusion with percutaneous coronary intervention (PCI), which clinically translated for a spectacular reduction in mortality and a decrease in the incidence of reinfarction, strokes and new cases of heart failure.

The aim of the study was to evaluate the association between selected baseline ECG parameters and LVR occurrence 6 months after a first STEMI treated with PCI.

## 2. Methods

### 2.1. Study Design

The study was a prospective cohort trial. All patients treated for a first STEMI using primary PCI with stent implantation in the Department of Cardiology at the Dr. Antoni Jurasz University Hospital in Bydgoszcz from 22 November 2005 to 31 December 2006 and from 22 February 2007 to 12 December 2008 were screened for participation in the study. The study was approved by the local Bioethics Committee of Collegium Medicum, Nicolaus Copernicus University in Toruń (ref. No. KB 440/2004). The following inclusion criteria were implemented: typical stenocardial pain with a duration of ≥30 min, time from the onset of symptoms to hospital admission <12 h, presence of electrocardiographic features suggestive of acute STEMI (ST-segment elevation of ≥0.2 mV in at least 2 adjacent leads for leads V_1_-V_3_ and/or ≥0.1 mV in at least 2 adjacent leads in the remaining leads, excluding lead aVR), informed consent for participation in the study. Exclusion criteria included previous MI, previous myocardial revascularization, cardiogenic shock on admission, advanced chronic cardiac failure (class III or IV according to New York Heart Association), left bundle branch block, isolated basal inferior (posterior) MI or isolated right ventricular infarction, permanent atrial fibrillation, hemodynamically significant valvular disease, primary cardiomyopathies, severe hypertension (≥180/110 mmHg) and/or creatinine concentration >2 mg/dL. All study participants underwent coronary angiography followed by immediate reperfusion with primary PCI. Interventional treatment and pharmacotherapy were applied in accordance with the guidelines of the European Society of Cardiology [16,17]. After PCI, detailed medical history was taken, and physical examination, standard laboratory tests, 12-lead electrocardiography and transthoracic echocardiography were performed in each patient. At hospital discharge a follow-up visit with transthoracic echocardiography was scheduled after 6 months. All patients granted written informed consent for participation in the study. The study was conducted in accordance with the Helsinki Declaration and the principles of Good Clinical Practice and was approved by the local bioethics commission.

### 2.2. Evaluation of Electrocardiograms

ECG recordings were performed at paper speed of 25 mm/s and 0.1 mV/mm calibration using 12 standard leads (I, II, III, aVR, aVL, aVF, V1–V6). Each ECG record was independently interpreted by two experienced cardiologists. In case of disagreement, a third cardiologist was consulted to determine the final interpretation. The following ECG parameters were evaluated:

- heart rate,
- location of MI,
- number of leads with ST-segment elevation,
- sum of ST-segment elevation in all leads,
- maximum ST-segment elevation in a single lead,
- presence of reciprocal ST-segment depression ≥0.1 mV on admission to hospital,
- number of leads with pathological Q-waves (according to the 2007 universal definition of MI) [18],
- the degree of ischemia according to the Birnbaum–Sklarovsky classification [2] and
- QRS complex width.

The location of STEMI was defined according to electrocardiographic criteria adopted in the 2007 universal definition of myocardial infarction, taking into account presence of ST-segment elevation in two contiguous leads among leads II, III, aVF for inferior wall location, leads V_1_–V_6_ for anterior wall location and leads I, aVL for lateral wall location [18].

### 2.3. Percutaneous Coronary Intervention

Coronary angiography and PCI procedures were performed with standard technique via the femoral artery, using an Integris Allura X-ray system (Philips, Amsterdam, the Netherlands). Adjuvant treatment with glycoprotein IIb/IIIa inhibitor (abciximab) and aspiration thrombectomy was used according to the operator’s discretion. Intracoronary stents were routinely used. Epicardial coronary flow was assessed according to the Thrombolysis In Myocardial Infarction (TIMI) score.

### 2.4. Concomitant Pharmacotherapy

At first medical contact, immediately after establishing the diagnosis of STEMI, all patients were pretreated with an intravenous bolus of unfractionated heparin (70 IU/kg, up to 5000 IU) and oral loading doses of clopidogrel (600 mg) and aspirin (300 mg). In the catheterization laboratory, a second weight-adjusted dose of unfractionated heparin was administered intraarterially. Throughout the hospitalization, aspirin and clopidogrel were continued (both 75 mg o.d.). Concomitant medications in all patients included perindopril and metoprolol, in doses adjusted for resting heart rate and blood pressure, and simvastatin (40 mg/day).

### 2.5. Echocardiography

LVR was assessed on the basis of two-dimensional transthoracic echocardiography performed with a Philips Sonos 7500 device (Philips, Andover, MA, USA) before hospital discharge and after 6 months. Acquisitions and measurements were made according to the recommendations of the American Society of Echocardiography and the European Association of Echocardiography [19,20]. LVR was defined, according to a previously validated definition [11,13], as an LVEDV increase of ≥20% within 6 months of STEMI. The inter- and intraobserver coefficients of variation for LVEDV assessed in the first 50 patients were below 5.0% and below 2.5%, respectively.

### 2.6. Statistical Analysis

Statistical analysis was performed using the Statistica 12.0 (StatSoft, Tulsa, OK, USA) and SPSS 23.0 (IBM, Armonk, NY, USA) software. Using the Shapiro–Wilk test, non-normal distribution of data was confirmed. Continuous variables were presented as medians with interquartile ranges, and non-parametric tests were used for statistical analysis. Comparisons between two groups were performed with the Mann–Whitney or Wilcoxon test when appropriate. Categorical variables were compared using the χ^2^ test for heterogeneity and Mantel–Haenszel test for linear trend. Two-sided *p*-value <0.05 was considered statistically significant.

In order to identify predictors for development of LVR, univariate and multivariate logistic regression models were used. Variables with a *p* value <0.1 in univariate analysis were subsequently introduced into the multivariate logistic regression models. In order to select the best fit, the stepwise backward regression method was applied. Subsequently, variables with no significant impact on the prevalence of LVR (*p* ≥ 0.05) were successively removed from the multivariate models according to their decreasing *p* values. Associations between the investigated variables and the likelihood of LVR were estimated using odds ratios (ORs) and their 95% confidence intervals (95% CIs). In order to identify factors associated with independent increase in LVEDV after 6 months of STEMI, a multiple linear regression analysis was performed. Best-fitting models were identified using backward stepwise regression. Variables with a *p* value of <0.1 in univariate analysis were introduced into the multiple linear regression model. Then, variables with no significant impact (*p* > 0.05) were removed one by one from the multivariate model. The relevant electrocardiographic variables were introduced individually (one at a time) as well as collectively (all together) to the created multivariate models, already containing demographic, clinical and angiographic parameters available early after admission. Finally, biochemical data were added to the models as they are available at later stages of hospitalization.

## 3. Results

### 3.1. The Course of the Study

Out of a total of 1297 patients meeting the inclusion criteria, 760 patients had at least one exclusion criterion, and 230 did not grant informed consent for participation in the study, most often due to expected difficulties in follow-up attendance. Eventually, 307 patients were included in the study. During the index STEMI hospitalization, 13 patients withdrew their consent for participation, and a further 14 were excluded from the analysis due to false diagnosis of STEMI (3 patients), poor quality of echocardiographic images precluding quantitative analysis (4 patients), incomplete ECG recordings (5 patients) and anticipated difficulties in follow-up attendance and echocardiographic assessment after 6 months (2 patients, including 1 patient due to dementia and 1 patient due to alcohol addiction syndrome). During the index hospitalization seven patients died: five due to cardiogenic shock, which developed after enrollment to the study, and two due to left ventricular free wall rupture. Seven patients did not attend the follow-up visit with echocardiographic assessment after 6 months, of whom six had died (five of cardiovascular causes and one due to a traffic accident), and one withdrew the consent for participation in the study. The final analysis included 249 patients.

### 3.2. Clinical, Demographic, Angiographic and Biochemical Parameters

The analyzed population predominantly consisted of men (74.7%). The median age was 57.0 years. Detailed demographic, clinical, angiographic and biochemical characteristics of the study population are presented in Table 1.

### 3.3. Echocardiographic Characteristics

Significant changes in parameters determining the size of the left ventricle were found 6 months after the index MI (Table 2). The median LVEDV increase was 11.0 (1.2-23.0) mL. The criterion for post-MI LVR, defined as an increase in LVEDV over 6 months by ≥20%, was met in 68 (27.3%) patients. Improvement in left ventricular systolic function after 6 months defined as an LVEF increase of ≥5% was found in 130 (52.2%) patients.

### 3.4. Electrocardiographic Characteristics

Detailed numerical data of baseline electrocardiographic parameters are presented in Table 3.

### 3.5. Comparison of Demographic, Clinical, Angiographic and Biochemical Characteristics of Patients with LVR (LVR (+) Group) and without LVR (LVR (−) Group)

There were no significant demographic or clinical differences between both groups. In addition, time from symptom onset to PCI was similar in patients with and without LVR at 6 months. In LVR (+) patients, the left anterior descending artery was significantly more common to be the infarct-related artery. The LVR (+) group also had worse TIMI flow in the infarct-related artery after PCI and presented more frequent use of GP IIb/IIIa receptor inhibitors. It was also in this group that higher releases of biochemical markers of myocardial necrosis and B-type natriuretic peptide at hospital discharge were found. Detailed numerical data are presented in Table 1. Importantly, there were no significant differences in discharge medications between patients with and without LVR at 6 months.

### 3.6. Electrocardiographic Characteristics of Patients with LVR

**Figure 1:**
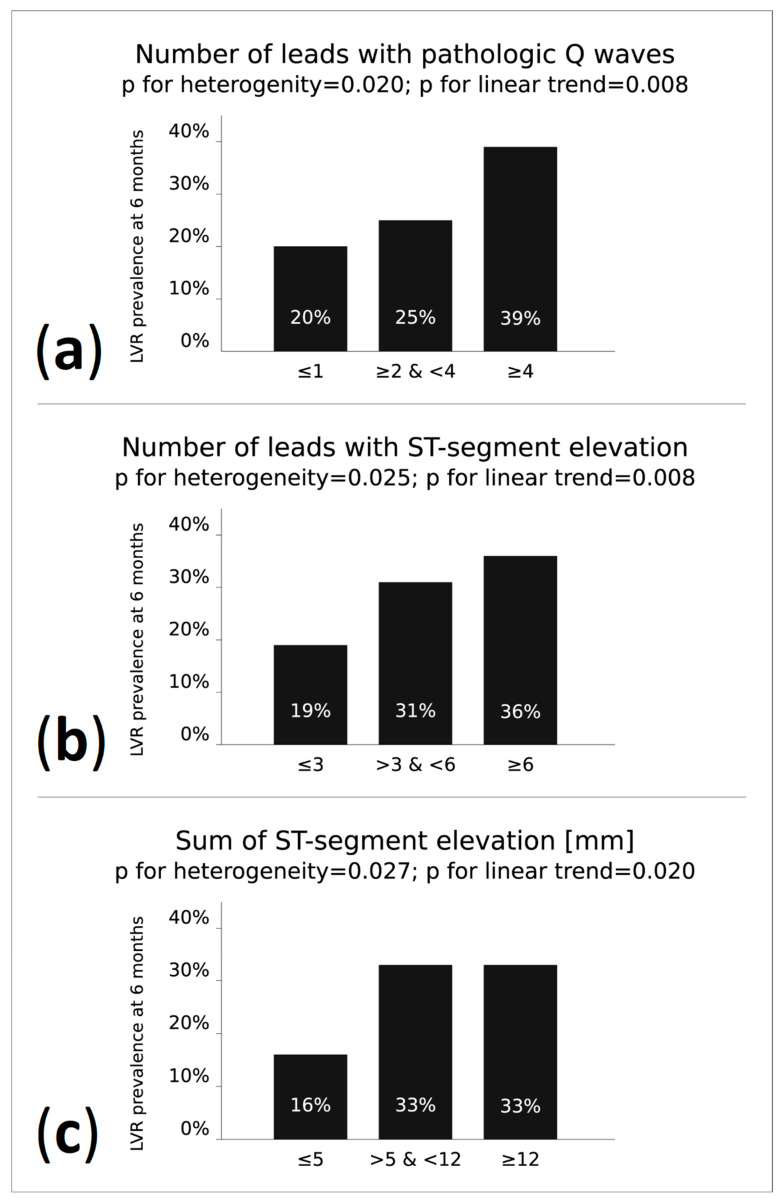
Prevalence of LVR at 6 months after hospital discharge after STEMI in patients classified according to increasing tertiles of (**a**) number of leads with pathological Q waves; (**b**) number of leads with ST-segment elevation and (**c**) sum of ST-segment elevation. LVR, left ventricular remodeling; STEMI, ST-segment elevation myocardial infarction.

In comparison with LVR (−) patients, the baseline electrocardiographic features seen in LVR (+) patients indicated a larger area of myocardial necrosis and a higher range and severity of ischemia (more leads with pathological Q waves, higher number of leads with ST-segment elevation, higher total ST-segment elevation). No differences regarding heart rate, the maximum ST-segment elevation, QRS complex duration, presence of reciprocal ST-segment depression ≥1mm and grade of ischemia according to Birnbaum and Sclarovsky were found on admission. There was a statistical trend toward more frequent anterior wall location of MI (*p* = 0.071) in the LVR (+) group. Detailed comparison of electrocardiographic data is presented in Table 3. Additionally, as presented in Figure 1, we found a significant heterogeneity and linear trend toward higher prevalence of LVR at 6 months with an increasing number of leads with pathological Q waves (OR for the upper vs. the lower tertile 2.60; 95% CI 1.28–5.32; *p* = 0.0076), increasing number of leads with ST-segment elevation (OR for the upper vs. the lower tertile 2.41; 95% CI 1.25–4.63; *p* = 0.0077) and a higher sum of ST-segment elevation (OR for the upper vs. the lower tertile 2.49; 95% CI 1.18–5.25; *p* = 0.0149).

### 3.7. Predictors of LVR Occurrence 6 Months after Discharge from Hospital in Univariate and Multivariate Logistic Regression Models

Together with investigated electrocardiographic parameters, we also considered demographic, clinical, angiographic and biochemical data from Table 1 as potential predictors of LVR. According to univariate logistic regression analysis, the baseline electrocardiographic predictors of LVR occurrence after 6 months included the number of leads with ST-segment elevation, total ST-segment elevation, maximum ST-segment elevation and number of leads with pathological Q wave. Additionally, the development of LVR 6 months after STEMI was also associated with increased body weight, left anterior descending artery (LAD) as the culprit vessel, poor flow in the infarct-related artery after PCI (TIMI <3), use of glycoprotein IIb/IIIa inhibitor, higher maximal concentration of cardiac troponin I, higher maximal activity of creatinine kinase MB isoenzyme and higher concentration of B-type natriuretic peptide at hospital discharge. The anterior location of MI showed a trend toward statistical significance (*p* = 0.073) in this aspect. The predictors of LVR occurrence 6 months after STEMI, presenting statistical significance (*p* < 0.05) or a trend towards it (0.05 ≤ *p* ≤ 0.10), in the univariate logistic regression analysis are shown inFigure 2.

**Figure 3:**
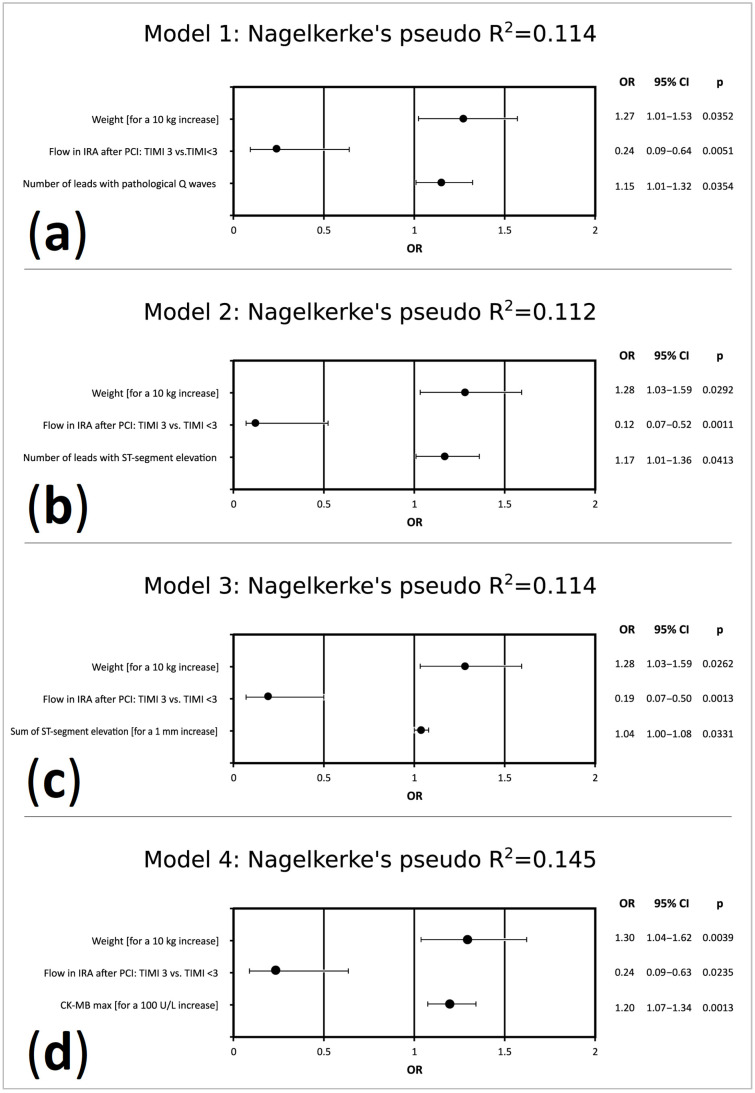
Predictors of LVR 6 months after STEMI in multivariate logistic regression models. Models (**a**–**c**) were created by adding a single electrocardiographic variable (**a**)—number of leads with pathological Q waves, (**b**)—number of leads with ST-segment elevation, (**c**)—sum of ST-segment elevation) to the demographic, clinical and angiographic data shown in Table 1. In model (**d**), biochemical data from Table 1 were also included. CI, confidence interval; CK-MB_max_, maximal activity of creatinine kinase MB isoenzyme; IRA, infarct-related artery; LVR, left ventricular remodeling; OR, odds ratio; STEMI, ST-segment elevation myocardial infarction; TIMI, Thrombolysis in Myocardial Infarction score.

Initially created multivariate logistic regression models incorporating a single electrocardiographic variable in addition to demographic, clinical and angiographic data available early after admission identified the number of leads with pathologic Q waves (Figure 3a), number of leads with ST-segment elevation (Figure 3b) and sum of ST-segment elevation (Figure 3c) as predictors of LVR. Then, we also included in multivariate logistic regression analysis biochemical data (creatine concentration, estimated glomerular filtration rate, glucose concentration, maximal concentration of cardiac troponin I, maximal activity of creatinine kinase MB isoenzyme, lipid parameters, concentration of B-type natriuretic peptide on hospital admission and at discharge). In the final model (Figure 3d), none of the analyzed electrocardiographic parameters, but increasing body weight, suboptimal or failed epicardial reperfusion (defined as TIMI flow after PCI <3) and higher maximal activity of creatinine kinase MB isoenzyme, was independently associated with the development of LVR 6 months after STEMI.

### 3.8. Determinants of Increase in LVEDV

In order to further evaluate the relationship between electrocardiographic parameters and the development of LVR, we applied multiple linear regression analysis with a backward elimination (Table 4). Initially, we aimed to determine which of the demographic, clinical, angiographic and electrocardiographic data available early after admission affected LVEDV increase over 6 months of follow-up. When single electrocardiographic parameters were added to the model, the number of leads with pathological Q waves (Model 1), number of leads with ST-segment elevation (Model 2) and sum of ST-segment elevation (Model 3) were associated with an increase in LVEDV. With collective inclusion of all electrocardiographic variables in the model, the number of leads with ST-segment elevation and number of leads with pathological Q waves were identified as independent predictors of LVEDV increase (Model 4). Finally, we also included in multiple linear regression analysis biochemical data (creatine concentration, estimated glomerular filtration rate, glucose concentration, maximal concentration of cardiac troponin I, maximal activity of creatinine kinase MB isoenzyme, lipid parameters, concentration of B-type natriuretic peptide on hospital admission and at discharge). In the final model (Model 5), none of the analyzed electrocardiographic parameters, but increasing patient’s height, suboptimal or failed epicardial reperfusion (defined as TIMI flow after PCI < 3) and higher maximal activity of creatinine kinase MB isoenzyme, was independently associated with an increase in LVEDV 6 months after STEMI.

## 4. Discussion

Our key observation is that the majority of the electrocardiographic parameters evaluated on admission to hospital carry a predictive value for LVR. In detail, several investigated electrocardiographic parameters (i.e., the number of leads with ST-segment elevation, number of leads with Q-waves and the sum of ST-segment elevation) were associated with the development of LVR 6 months after STEMI in both univariate analysis and multivariate analysis including demographic, clinical and angiographic data. However, after adding biochemical parameters, particularly biomarkers of myocardial necrosis, to multivariate analysis models, the analyzed electrocardiographic parameters lost their predictive value for LVR.

Importantly, the present study was conducted in a homogeneous group of patients, in terms of the form of ACS presentation (patients with a first STEMI) and the applied reperfusion treatment (primary PCI). This group homogeneity, together with strict adherence to inclusion and exclusion criteria, eliminated many potential confounders and makes it possible to refer the results to patients treated in accordance with current guidelines. In the following discussion, the significance of selected ECG parameters with regard to the obtained results and available literature data is discussed.

In 1998, Murkofsky et al. conducted a study involving 226 patients referred for radionuclide ventriculography. They found that the width of QRS complexes >100 ms was associated with a significant increase in left ventricular end-systolic volume (LVESV) and LVEDV. There were also moderate, positive correlations between the width of QRS complexes and both LVESV and LVEDV and a negative correlation between the QRS duration and LVEF [14]. According to our research, QRS complex width on admission had no predictive value regarding LVR after 6 months after STEMI. This finding contradicts data from the literature indicating a positive association between QRS complex width and the occurrence of LVR [14]. This inconsistency may be potentially explained by different clinical settings (STEMI patients vs. subjects with suspected stable coronary artery disease).

Numerous reports associating the presence of pathological Q waves with more frequent death, heart failure and cardiogenic shock can be found in the literature [3,5,21]. However, there are no publications regarding the relationship of pathological Q waves with LVR. The appearance of new Q waves during MI is associated with a larger area of necrosis and higher mortality compared with MI without Q waves. Huey et al. demonstrated that conservatively treated patients with Q-wave MI had less frequently preserved patency of the infarct-related artery than patients with non-Q-wave MI (24% vs. 57%; *p* = 0.001). Moreover, patients with Q-wave MI presented a higher creatine kinase activity at the 4th hour of MI (1372 ± 964 vs. 664 ± 924 U/L; *p* = 0.0002) and lower LVEF (46 ± 12% vs. 54 ± 9%; *p* = 0.0003) [3]. In a study by Armstrong et al., in patients with acute MI treated with PCI, the presence of Q waves on admission, as compared with non-Q-wave MI patients, was associated with a 2.5-fold increase in mortality and the occurrence of a composite endpoint including death, heart failure and cardiogenic shock in a 90-day observation. The presence of Q waves was a better predictor of 90-day mortality than the time from the onset of MI pain to PCI [21]. Similar results were obtained in the APEX-AMI study, including patients with STEMI treated with PCI. The presence of Q waves in leads with ST-segment elevation in the initial ECG proved to be a better predictor of death and the composite end point (death, cardiogenic shock and heart failure) in a 90-day follow-up than the time from onset of symptoms to PCI [4]. In the PLATO study, a higher annual overall mortality was observed in patients with Q waves present on admission compared with patients without Q waves. In multivariate regression, taking into account age, heart rate, systolic pressure, Killip–Kimball class and MI location, it was the presence of Q waves on admission, not the time from the onset of symptoms, that was an independent predictor of annual overall mortality due to vascular causes [5]. In our study, the occurrence of LVR 6 months after STEMI was associated with the presence of pathological Q waves in a significantly larger number of leads than in patients without LVR after 6 months. In the univariate regression model, the number of leads with pathological Q waves on admission was a significant predictor of LVR (OR 1.21; 95% CI 1.07–1.37; *p* = 0.0033). Additionally, the number of leads with pathological Q waves on admission was also a predictor of both LVR and increase in LVEDV in multivariate models, but only if laboratory biomarkers were not included in the analysis. When arranged into tertiles, particularly the third tertile (≥4 leads with pathological Q-waves) was associated with a marked increment in LVR prevalence.

In a study by Manes et al. involving 272 patients with anterior wall MI, mainly treated with fibrinolysis (87%), the sum of ST elevation, maximum ST-segment elevation and number of leads with ST-segment elevation ≥1 mm were independent predictors of the occurrence of left ventricle enlargement after 90 days. At hospital discharge, the presence of ST-segment elevation of ≥1 mm in each subsequent lead corresponded to an increase in the volume of the left ventricle by 3.5 mL (95% CI 1.6–5.5 mL; *p* < 0.0001) [15]. In a study using magnetic resonance, Husser et al. proved that the sum of ST-segment elevation is the best predictor of coronary microcirculation obstruction in STEMI patients treated with PCI. The same study demonstrated that patients with obstructed coronary microcirculation, when compared with those with preserved microcirculation, had higher mortality and post-MI LVR rates. They also presented higher values of left ventricular end-systolic volume index (LVESVI) (54 vs. 29 mL/m^2^; *p* < 0.0001) and left ventricular end-diastolic volume index (LVEDVI) (93 vs. 70 mL/m^2^; *p* < 0.0001), both being recognized as indicators of LVR occurrence [22].

In our research, three parameters related to ST-segment elevation were analyzed: the number of leads with ST-segment elevation, the sum of ST-segment elevations and the amplitude of maximum ST-segment elevation in a single lead. All of them were associated with LVR occurrence in the univariate regression models. Moreover, multivariate models, including all data obtained on hospital admission or during primary PCI (performed just after admission), showed that the number of leads with ST-segment elevation and the sum of ST-segment elevations were electrocardiographic predictors of LVR and LVEDV increase after 6 months of STEMI. When analyzed as tertiles, it showed that a particularly high prevalence of LVR (exceeding 30%) occurred in the second and third tertile, corresponding to >3 leads with ST-segment elevations and a sum of ST-segment elevations >5 mm. On the other hand, ECG parameters failed to show independent predictive value for LVR in our study when considered together with laboratory biomarkers.

Some limitations of this study should be mentioned. Firstly, the relatively well-preserved left ventricular systolic function and a relatively low risk of death and cardiovascular complications seen in the study group due to the applied exclusion criteria, in conjunction with relatively early application of reperfusion therapy, do not allow extrapolation of the obtained results to all STEMI patients. More studies in heterogeneous STEMI patients are needed before unrestricted application of our findings to the world-wide population can be made. Secondly, the endpoint (LVR) assessed in the study, despite a well-documented association with clinical endpoints, is only a surrogate endpoint, as this study did not have adequate power to assess clinical endpoints. Additionally, clinical follow-up in our study is limited to 6 months. Thirdly, the study population is a small portion of initially available cohort of patients, and recruitment for the study was conducted in years 2005–2008. The period that elapsed from recruiting patients to the project and related changes in clinical practice could have a potential impact on the results. Fourthly, the use of magnetic resonance imaging instead of echocardiography would allow more accurate assessment of the left ventricle and LVR, and provide even more reliable information on the extent of myocardial necrosis and obstruction of coronary microcirculation. Fifthly, we did not consider left ventricular mass as an indicator of LVR. Finally, we did not assess post-reperfusion ECG parameters.

## 5. Conclusions

Our study demonstrates modest utility of baseline ECG for the prediction of LVR occurrence after six months of STEMI.

## Figures and Tables

**Figure 2:**
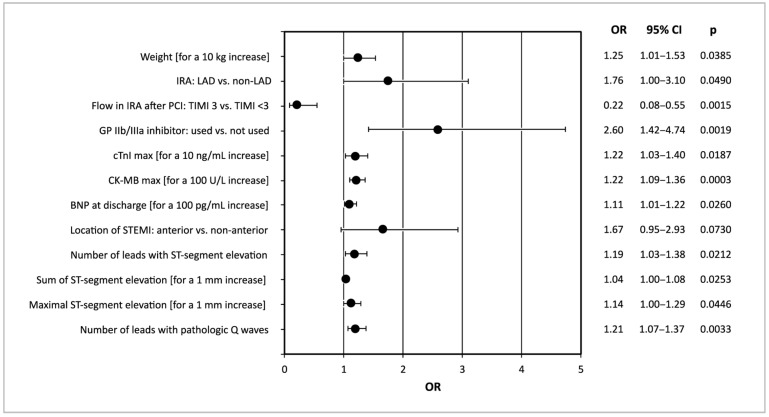
Demographic, clinical, angiographic, biochemical and electrocardiographic predictors of LVR 6 months after STEMI in univariate logistic regression analysis. BNP, B-type natriuretic peptide; CI, confidence interval; CK-MB_max_, maximal activity of creatinine kinase MB isoenzyme; cTnI_max_, maximal concentration of cardiac troponin I; IRA, infarct-related artery; LVR, left ventricular remodeling; OR, odds ratio; PCI, percutaneous coronary intervention; STEMI, ST-segment elevation myocardial infarction; TIMI, Thrombolysis in Myocardial Infarction score.

**Table 1:** Demographic, clinical, angiographic and biochemical characteristics of the study population (median (lower quartile-upper quartile) or number (percent)).

Variable	Overall Study Population (*n* = 249)	Patients with LVR at 6 Months (*n* = 68)	Patients without LVR at 6 Months (*n* = 181)	*p*-Value *
Age (years)	57.0 (51.0-64.0)	55.5 (50.0-62.0)	58.0 (52.0-64.0)	0.166
Gender (male/female)	186 (74.7%)/63 (25.3%)	52 (76.5%)/16 (23.5%)	134 (74.0%)/47 (26.0%)	0.693
Weight (kg)	78.0 (70.0-88.0)	80.0 (71.0-91.5)	78.0 (70.0-87.0)	0.092
Height (cm)	170.0 (165.0-176.0)	172.5 (166.0-176.5)	170.0 (164.0-176.0)	0.085
Time from symptom onset to PCI (min)	220.0 (150.0-331.5)	228.0 (140.0-345.0)	220.0 (156.0-324.5)	0.908
**Risk factors for coronary artery disease**				
BMI (kg/m^2^)	26.8 (24.2-29.4)	26.9 (24.7-30.0)	26.3 (24.2-29.1)	0.252
Hypertension	103 (41.4%)	31 (45.6%)	72 (39.8%)	0.407
Diabetes mellitus	50 (20.1%)	16 (23.5%)	34 (18.8%)	0.405
Previously known hyperlipidemia	58 (23.3%)	14 (20.6%)	44 (24.3%)	0.536
Current or ex-smoker	164 (65.9%)	44 (64.7%)	120 (66.3%)	0.813
Positive family history of IHD	61 (24.5%)	61 (24.5%)	42 (23.2%)	0.439
**Angiographic characteristics**				
IRA: LAD/non-LAD	121 (48.6%)/128 (51.5%)	40 (58.8%)/28 (41.2%)	81 (44.8%)/100 (55.2%)	0.048
TIMI 0 flow in IRA before PCI	144 (57.8%)	45 (66.2%)	99 (54.7%)	0.102
TIMI 3 flow in IRA after PCI	229 (92.0%)	56 (82.4%)	173 (95.6%)	0.0006
Multivessel coronary artery disease	143 (57.4%)	41 (60.3%)	102 (56.4%)	0.5753
Stent implantation	245 (98.4%)	66 (97.1%)	179 (98.9%)	0.305
GP IIb/IIIa inhibitor usage	66 (26.5%)	28 (41.2%)	38 (21.2%)	0.0016
**Biochemical characteristics**				
Creatinine on admission (mg/dL)	0.90 (0.81-1.06)	0.93 (0.88-1.05)	0.90 (0.80-1.06)	0.618
eGFR calculated using the CKD-EPI formula (mL/min/1.73 m^2^)	84.4 (74.1-94.5)	84.4 (74.6-94.6)	84.7 (73.4-94.5)	0.869
Glucose on admission (mg/dL)	138.5 (122.0-169.0)	140.0 (122.0-170.0)	136 (120.0-169.0)	0.376
cTnI_max_ (ng/mL)	41.2 (11.8-50.0)	50.0 (20.7-50.0)	35.7 (10.5-50.0)	0.012
CK-MB_max_ (U/L)	242.0 (116.5-414.0)	375.5 (162.0-612.0)	207.0 (97.5-375.0)	0.0002
Total cholesterol (mg/dL)	223.0 (195.0-251.0)	221.5 (196.0-244.0)	224.0 (195.0-253.0)	0.582
LDL-C (mg/dL)	145.0 (125.0-173.0)	146.5 (123.5-166.0)	144.5(126.0-176.0)	0.771
HDL-C (mg/dL)	52.0 (46.0-59.0)	53.0 (48.0-61.0)	51.0(45.0-58.0)	0.205
Triglycerides (mg/dL)	82.0 (59.0-128.0)	85.0 (57.0-130.5)	82.0(60.0-124.0)	0.729
BNP on admission (pg/mL)	53.9 (27.9-106.5)	51.5 (26.8-102.2)	55.3(28.6-114.6)	0.854
BNP at discharge (pg/mL)	139.8 (74.7-284.2)	159.5 (82.5-396.4)	125.9 (70.1-239.4)	0.021
**Medications prescribed at hospital discharge**				
Acetylsalicylic acid	249 (100.0%)	68 (100.0%)	181 (100.0%)	N/A
Clopidogrel	249 (100.0%)	68 (100.0%)	181 (100.0%)	N/A
Statin	249 (100.0%)	68 (100.0%)	181 (100.0%)	N/A
Beta blocker	247 (99.2%)	67 (98.5%)	180 (98.9%)	0.6772
ACEI or ARB	249 (100.0%)	68 (100.0%)	181 (100.0%)	N/A
Aldosterone antagonist	25 (10.0%)	8 (11.8%)	17 (9.4%)	0.5702
Diuretic	24 (9.6%)	9 (13.2%)	15 (8.3%)	0.3484

ACEI, angiotensin-converting enzyme inhibitor; ARB, angiotensin II receptor blocker; BMI, body mass index; BNP, B-type natriuretic peptide; CK-MB_max_, maximal activity of creatinine kinase MB isoenzyme; CKD-EPI, Chronic Kidney Disease Epidemiology Collaboration; cTnI_max_, maximal activity of troponin I; eGFR, estimated glomerular filtration rate; HDL-C, high-density-lipoprotein cholesterol; IHD, ischemic heart disease; IRA, infarct-related artery; LAD, left anterior descending artery; LDL-C, low-density-lipoprotein cholesterol; LVR, left ventricular remodeling; N/A, not applicable, PCI, percutaneous coronary intervention; TIMI, Thrombolysis in Myocardial Infarction score.* For comparison between groups with and without LVR.

**Table 2:** Echocardiographic characteristics of the overall study population (median (lower quartile-upper quartile) or number (percent)).

Variable	At Discharge (*n* = 249)	At 6 Months after Discharge (*n* = 249)	*p*-Value
LA (mm)	40.0 (37.0-43.0)	40.0 (38.0-44.0)	0.000006
LVEDd (mm)	49.0 (45.0-53.0)	50.0 (46.0-54.0)	<0.000001
LVESd (mm)	34.0 (30.0-37.0)	34.0 (31.0-37.0)	0.038
LVEDV (mL)	99.4 (84.0-121.0)	110.0 (94.0-134.0)	<0.000001
LVESV (mL)	55.0 (45.0-69.0)	57.0 (48.0-76.0)	<0.000001
LVEF (%)	44.0 (39.0-48.4)	46.0 (42.0-51.5)	<0.000001
LVSD (LVEF ≤ 40%)	84.0 (33.7%)	52.0 (20.9%)	<0.00001
WMSI (points)	1.56 (1.38-1.75)	1.44 (1.31-1.69)	<0.000001

LA, left atrium end-systolic diameter; LVEDd, left ventricular end-diastolic diameter; LVEDV, left ventricular end-diastolic volume; LVEF, left ventricular ejection fraction; LVESd, left ventricular end-systolic diameter; LVESV, left ventricular end-systolic volume; LVSD, left ventricular systolic dysfunction; WMSI, wall motion score index.

**Table 3:** Baseline electrocardiographic characteristics of the study population (median (lower quartile-upper quartile) or number (percent)).

Variable	Overall Study Population (*n* = 249)	Patients with LVR at 6 Months (*n* = 68)	Patients without LVR at 6 Months (*n* = 181)	*p*-Value *
Heart rate (BPM)	75.0 (62.0-88.0)	79.0 (64.5-89.0)	74.0 (62.0-86.0)	0.197
Anterior location of STEMI	116 (47.0%)	38 (55.9%)	78 (43.1%)	0.071
Number of leads with ST-segment elevation (n)	4.0 (3.0-6.0)	5.0 (3.0-6.0)	4.0 (3.0-6.0)	0.019
Sum of ST-segment elevation (mm)	8.5 (4.0-14.0)	9.5 (6.3-16.0)	8.0 (4.0-13.5)	0.014
Maximal ST-segment elevation (mm)	3.0 (2.0-4.0)	3.0 (2.0-5.0)	3.0 (1.8-4.0)	0.089
Number of leads with pathological Q waves (n)	2.0 (1.0-4.0)	3.0 (1.5-5.0)	2.0 (1.0-3.0)	0.004
Presence of reciprocal ST-segment depression ≥1mm	193 (77.5%)	50 (73.5%)	143 (79.0%)	0.356
QRS duration (ms)	95.0 (85.0-100.0)	93.0 (85.0-100.0)	96.5 (87.5-102.0)	0.170
Grade of ischemia according to Birnbaum and Sclarovsky	grade 2: 198(79.5%); grade 3: 51(20.5%)	grade 2: 53 (77.9%); grade 3: 15 (22.1%)	grade 2: 145 (80.1%); grade 3: 36 (19.9%)	0.705

BPM, beats per minute; LVR, left ventricular remodeling; PCI, percutaneous coronary intervention. * For comparison between groups with and without LVR.

**Table 4:** Impact of demographic, clinical, angiographic, biochemical and electrocardiographic variables on increase in LVEDV 6 months after STEMI in multiple linear regression models. Models 1–3 were obtained by adding a single electrocardiographic variable to the demographic, clinical and angiographic data shown in Table 1. In the subsequently built Model 4, we included all electrocardiographic variables. Finally, we added biochemical data presented in Table 1 to previously analyzed demographic, clinical, angiographic and electrocardiographic variables (Model 5).

Variable	Beta Coefficient	Beta Coefficient Standard Error	Direction Component Beta	Direction Component Beta Standard Error	*p*-Value
*Model 1. Characteristics: R = 0.428; R^2^ = 0.183; corrected R^2^ = 0.173; p < 0.0001*					
Intercept			19.40	4.47	<0.0001
IRA: LAD/non-LAD	0.18	0.06	6.73	2.30	0.0037
Flow in IRA after PCI: TIMI 3 vs. TIMI < 3	−0.23	0.06	−15.91	4.09	0.0001
Number of leads with pathological Q waves	0.22	0.06	1.83	0.54	0.0007
*Model 2. Characteristics: R = 0.404; R^2^ = 0.163; corrected R^2^ = 0.153; p < 0.00001*					
Intercept			−33.42	24.45	0.1729
Height (cm)	0.13	0.06	0.32	0.14	0.0284
Flow in IRA after PCI: TIMI 3 vs. TIMI < 3	−0.29	0.06	−19.91	4.05	<0.0001
Number of leads with ST-segment elevation	0.24	0.06	2.34	0.59	0.0001
*Model 3. Characteristics: R = 0.417; R^2^ = 0.174; corrected R2 = 0.160; p < 0.00001*					
Intercept			−26.80	24.37	0.2725
Height (cm)	0.12	0.06	0.30	0.14	0.0366
IRA: LAD/non-LAD	0.17	0.07	6.42	2.46	0.0097
Flow in IRA after PCI: TIMI 3 vs. TIMI < 3	−0.28	0.06	−19.30	4.05	<0.0001
Sum of ST-segment elevation (for a 1 mm increase)	0.13	0.06	0.32	0.16	0.0422
*Model 4. Characteristics: R = 0.484; R^2^ = 0.235; corrected R^2^ = 0.220; p < 0.00001*					
Intercept			−39.04	25.90	0.1332
Height (cm)	0.14	0.06	0.34	0.15	0.0260
Flow in IRA after PCI: TIMI 3 vs. TIMI < 3	−0.29	0.06	−19.98	4.32	<0.0001
Number of leads with ST-segment elevation	0.18	0.06	1.77	0.65	0.0065
Number of leads with pathological Q waves	0.20	0.07	1.74	0.58	0.0029
*Model 5. Characteristics: R = 0.471; R^2^ = 0.222; corrected R^2^ = 0.209; p < 0.00001*					
Intercept			−36.42	23.81	0.1275
Height (cm)	0.13	0.06	0.33	0.14	0.0194
Flow in IRA after PCI: TIMI 3 vs. TIMI < 3	−0.24	0.06	−16.53	3.97	<0.0001
IRA: LAD/non-LAD	0.16	0.06	5.97	2.23	0.0079
CK-MB_max_ (for a 100 U/L increase)	0.27	0.06	1.94	0.43	<0.0001

CK-MB_max_, maximal activity of creatinine kinase MB isoenzyme; IRA, infarct-related artery; LAD, left anterior descending artery; LVEDV, left ventricular end-diastolic volume; PCI, percutaneous coronary intervention; STEMI, ST-segment elevation myocardial infarction; TIMI, Thrombolysis in Myocardial Infarction score.

## Data Availability

Data sharing is not applicable to this article.
